# The Process From Turnover Intention to Turnover Decision in Chinese Nurses With Master's Degree: A Grounded Theory Study

**DOI:** 10.1155/jonm/6574378

**Published:** 2025-08-24

**Authors:** Na Xu, Xiumei Wang, Wenxian Wu, Yanbin Niu, Ying Wang, Wanling Li

**Affiliations:** ^1^Department of Nursing, Shanxi Bethune Hospital, Shanxi Academy of Medical Sciences, Third Hospital of Shanxi Medical University, Tongji Shanxi Hospital, Taiyuan 030032, China; ^2^Nursing Department, Tongji Medical College, Huazhong University of Science and Technology, Wuhan 430030, China; ^3^Department of Geriatrics, Tongji Hospital of Tongji Medical College, Huazhong University of Science and Technology, Wuhan 430030, China

**Keywords:** constructivist grounded theory, master's degree, nurse, qualitative research, turnover decision, turnover intention

## Abstract

**Aim:** This study explores the stages from turnover intention to turnover decision-making among Chinese nurses with a master's degree, aiming to identify the enablers and barriers influencing turnover decisions.

**Design:** A constructivist grounded theory approach was adopted to develop a theoretical framework for understanding turnover decision-making among master's degree nurses.

**Methods:** Through purposive and theoretical sampling, nurses with a master's degree who had either left their positions or expressed a high turnover intention within the past 2 years were selected. Semistructured interviews were conducted face to face or online from May to August 2023. Participants were employed or had previously worked in tertiary hospitals across Chinese provincial capitals. Theoretical sampling and constant comparative analysis were employed to achieve theoretical saturation. Data collection and analysis occurred concurrently, with Charmaz's constructivist grounded theory applied for continuous comparison to identify core categories.

**Results:** A total of 18 nurses with master's degrees were recruited from tertiary hospitals in regions including Shanxi, Shaanxi, Shanghai, Shandong, and Tianjin. The process from turnover intention to decision is influenced by four key factors: triggers, fuels, retardants, and accelerants. This progression spans four stages: intention generation, turnover latency, turnover deliberation, and final decision.

**Conclusions:** Nursing managers can identify and track the progression of turnover intention through the four stages from intention to decision. Tailored management strategies should focus on key influencing factors, strengthening protective factors that impact turnover decisions, helping nurses rebuild confidence in continued employment, and reducing both turnover intention and overall turnover rates.

## 1. Introduction

As the global demand for healthcare services grows and the field evolves rapidly, nursing roles are becoming increasingly specialized and complex [1]. The differentiation of nursing skills at various levels is crucial in addressing the gap between nursing needs and societal demands, as well as advancing nursing professionalization [2–4]. In response to these demands, many high-income countries have integrated with advanced education, such as those holding master's degrees, into critical clinical roles. In the United Kingdom, advanced nurse practitioners (ANPs) are empowered to independently diagnose, prescribe, and manage patient care [5]. Similarly, in Canada and Australia, these nurses play key roles in primary care, rural health, and chronic disease management [6, 7].

Reflecting this global trend, the number of nurses holding master's degrees and engaged in clinical practice has been steadily rising in recent years [8, 9]. In the United States, registered nurses with advanced education and training at the national level, holding a master's or doctoral degree, are classified as advanced practice registered nurses (APRNs) [10]. Currently, over 133,000 APRNs are actively practicing in the United States, with substantial numbers entering the workforce each year [11, 12]. Nevertheless, projections indicate that by 2025, the supply of APRNs in the United States will still fall short of demand by approximately 20% [13]. In China, clinical nurses with master's degrees—though not formally designated as APNs or APRNs—often take on similar advanced clinical roles. The proportion of registered nurses with master's degrees has increased from 0.1% in 2012 to 0.3% in 2022 [14]. In 2024, nurses with master's degrees accounted for 0.71% of registered nurses in tertiary hospitals [15]. Research indicates that nurses with advanced education, having received systematic training, are vital in driving healthcare innovation and improving care delivery [8]. A study in the Lancet found that adequate staffing and higher nursing education levels can reduce patient mortality by nearly 30% [16]. Despite their critical role, however, nurses with master's degrees face high turnover rates.

Turnover intention refers to an employee's deliberate consideration of leaving an organization, encompassing psychological, cognitive, and behavioral dimensions that evolve through distinct phases [17, 18]. Globally, high turnover rates among nurses with advanced education threaten healthcare continuity and exacerbate workforce shortages [19]. A 2012 report by Cejka Search and the American Medical Group Association found that the turnover rate for APRNs was 12.6% in 2011 [20]. Additionally, a study by Poghosyan et al. reported a 22% turnover intention among ANPs in 2022 [21]. Similarly, in China, nurses with master's degrees exhibit strong turnover intentions yet receive minimal attention in research [9]. For instance, at a prominent nursing college in China, of the six master's degree nurses who graduated in 2022 and chose to work in hospitals, two had already left by 2023, resulting in a turnover rate of 33.33%. This trend is prevalent across Chinese tertiary hospitals. In a tertiary hospital in Shanxi Province, 7 master's degree nurses were recruited in 2020 and 18 in 2021, with turnover rates of 42.86% and 38.88%, respectively, as of November 1, 2023. These figures reflect local data, as official national statistics are not yet available. Tian et al. found that turnover intention escalates following negative events, ultimately leading to turnover [22]. High turnover among master's degree nurses disrupts nursing research and innovation, undermining workforce stability and efficiency [23]. Therefore, understanding the turnover intentions and their influencing factors for these nurses—and intervening at the intention stage—is crucial for reducing turnover rates and stabilizing the nursing workforce.

Current research on nursing turnover, both domestically and internationally, primarily focuses on specialized departments and high-risk groups such as male nurses, rotating nurses, new nurses, and those with low tenure [24, 25]. While studies on turnover among nurses with master's degrees exist, they predominantly utilize quantitative methods to identify factors influencing turnover intentions, such as job expectations, professional burnout, and job satisfaction [8, 9, 26, 27]. However, turnover decision-making is a dynamic, multistage process shaped by various subjective factors [28, 29]. Quantitative methods are insufficient for capturing the full complexity of this process, particularly the dynamic and evolving interplay of factors over time [30, 31]. Consequently, existing studies do not provide a comprehensive understanding of the process from turnover intention to decision-making among Chinese nurses with master's degrees. Furthermore, beyond the fixed elements of job characteristics and salary, it remains unclear whether personal traits, educational methods in universities, and perceived professional environments [32] significantly influence turnover decisions among this group.

Few studies have investigated the factors and dynamic process of turnover decision-making in this specific group. This study aims to fill this gap by employing grounded theory to explore the stages from turnover intention to the actual decision-making process among nurses with master's degrees. This approach will offer a deeper understanding of the factors that either promote or inhibit their decision to leave. Ultimately, it aims to enable stakeholders and policymakers to detect early signs of turnover, identify modifiable factors influencing turnover decisions, and provide a theoretical foundation for refining talent development programs and management strategies for these nurses.

## 2. Methods

### 2.1. Design

This study employed Charmaz's constructivist grounded theory methodology [33] to generate a substantive theory that explains the underlying factors and dynamic process of turnover decision-making among nurses with master's degrees, from turnover intention to actual departure. Grounded theory, rooted in symbolic interactionism, aims to explain phenomena by emphasizing the interaction between individuals and their social contexts. Charmaz introduces the concept of theory generation, advocating for flexible application of grounded theory methods to acknowledge the researcher's role and the ways, in which theories emerge within specific social and power contexts [34]. This methodology effectively captures the interaction between individual psychological factors (e.g., job burnout) and organizational structural factors (e.g., regulatory frameworks) by analyzing the past and present experiences of nurses with master's degrees. It highlights the nonlinear nature of the decision-making process, offering valuable insights for the development of theories regarding nursing turnover decisions [35, 36]. This report adheres to the Consolidated Criteria for Reporting Qualitative Research (COREQ) [37].

### 2.2. Setting and Sample

The study was conducted in provincial tertiary hospitals in China, where nurses with master's degrees exhibit a high propensity to leave [9]. These nurses are required to rapidly develop clinical, scientific, and managerial competencies but often struggle to integrate them into clinical practice due to a lack of clinical experience. Additionally, they face challenges such as lower salaries, older age, and the pressure of balancing multiple roles—personal relationships, family obligations, work, and childbearing.

Purposeful and theoretical sampling was employed to select nurses with master's degrees who had either resigned or currently worked in Chinese tertiary hospitals and exhibited a high turnover intention. Before inclusion, turnover intention was assessed using the Turnover Intention Scale developed by Michael and Spector [38]. This scale, widely used to assess turnover intention among nurses [39, 40], has a total score range of 6–24, with higher scores indicating stronger turnover intention. A score above 12 is considered indicative of a high level of turnover intention [23].

The principal investigators, N.X. and W.W., both nurses with master's degrees, are well-versed in the Chinese hospital management system, daily operations, and the specific circumstances faced by nurses with master's degrees in China. Initially, purposive sampling was used to select participants from among the lead investigators' colleagues [41], resulting in the inclusion of 11 participants based on predefined criteria. Subsequently, theoretical sampling was employed to recruit additional participants who had either resigned or were currently employed in tertiary hospitals across various provinces in China. Seven more participants were recruited using theoretical sampling principles [42]. This sampling method allows researchers to capture the impact of incidental events, reflect on deeper connections within the development of concepts, and ensure that the research findings cover a broad range of situations. Thus, it contributes to a richer theoretical understanding of the process from turnover intention to turnover decision-making among nurses with master's degrees [42].

The study included nurses with master's degrees who had either resigned or exhibited high turnover intention but had not yet left their positions. Inclusion criteria were as follows: (1) full-time master's degree nurses; (2) possession of a valid nurse practice certificate from the People's Republic of China; (3) at least six months of cumulative clinical work experience; (4) either voluntarily resigned within the past two years and completed formal resignation procedures or were still employed with high turnover intention as measured by a score of ≥ 12 on the Turnover Intention Scale; and (5) voluntary participation in the study. Nurses who were contracted with specific work units and received with state-sponsored training expenses were excluded, as these nurses were required to fulfill service obligations and could not make independent decisions regarding resignation until their service term concluded.

The recruitment process involved two researchers (N.X. and W.W.) sending email invitations to prospective participants, which included essential information about the study's purpose, objectives, data collection methods, data management plan, and consent form. After conducting interviews and analyzing data from 18 nurses, it was determined that no new information emerged beyond this sample. Thus, the final participant count was 18 nurses.

### 2.3. Data Collection

Between May and August 2023, individual semistructured in-depth interviews were conducted to explore participants' experiences and the process they underwent from turnover intention to turnover decision. Prior to the interviews, the format, time, and location were arranged according to the participants' convenience. Interviewees were selected from diverse regions across China. Face-to-face interviews were held with participants from nearby locations, while online interviews were conducted with those from other provinces due to geographical constraints.

Interviews were scheduled at mutually convenient times to ensure a distraction-free environment, essential for clear audio capture and maintaining conversational flow. All interviews were conducted by three interviewers (N.X., W.W., and W.L.) to ensure consistency. Given that all participants were native Mandarin speakers, interviews were conducted in Mandarin. The duration of the interviews ranged from 28 to 70 min. Digital recorders and field notes were used to capture observations regarding participants' emotions, feelings, and behaviors during the interviews. All interview records were transcribed verbatim on the day of each interview. Afterward, the preliminary findings were shared with four participants—two from the same province as the primary researcher *via* WeChat and two from other provinces *via* email. Participants were asked to provide feedback by phone within 3 days, ensuring that the interpretations accurately reflected their experiences.

An interview guide was developed, containing key open-ended questions informed by previous research [22] and preliminary interviews. These questions included the following: (1) What events or reasons led you to consider turnover? Please describe your experiences and emotions during that time. (2) How did your willingness to leave evolve from initial consideration to remaining in your position or deciding to resign? What factors made you more inclined to leave? (3) Can you describe a specific experience that made you reconsider leaving? (4) What factors do you believe ultimately led to your decision to leave?

In line with theoretical sampling, participants' expressions and responses guided follow-up questions throughout the interviews. Initially, data were collected from varied locations, individuals, and events to explore attributes related to the theme, forming conceptual dimensions. Based on preliminary analyses and evolving concepts, new interview entry points were selectively determined, focusing on the theme and the ongoing development of concepts. This process ensured the identification of appropriate interviewees to meet the emerging data collection needs. As interviews progressed, scattered concepts were connected and refined along the narrative of turnover decision-making, leading to a preliminary theoretical explanation. Sampling continued until theoretical saturation was reached, at which point no new relevant concepts emerged, and data collection ceased.

### 2.4. Data Analysis

NVIVO 12.0 software was employed for the initial coding, focused coding, theoretical coding, and continuous comparison of interview data, with the goal of forming categories or concepts and exploring their interconnections [43]. The data analysis followed these steps: Initial Coding: Data were coded line by line to gain familiarity with it and identify potential categories and processes related to turnover decisions among nurses with master's degrees [33]. Each coding unit (such as a line or paragraph) was required to clearly express a distinct idea or behavior for inclusion. Focused Coding: Based on the initial coding, core categories were identified in the data. The selection of core categories was guided by their frequency and significance, clustering similar codes together. The aim of focused coding was to reduce data complexity, concentrating on major categories while ensuring the analysis remained grounded in the data. Theoretical Coding: Building upon focused coding, a deeper analysis of identified core categories was conducted to achieve saturation. Through constant comparison, the analysis became more interpretive and analytical, with the goal of developing concepts and emerging theories [34]. The criteria for theoretical coding were met when no new information or categories emerged from the data, signaling saturation.

Data analysis was primarily conducted by N.X. and W.W., who held regular meetings and discussions to ensure consistency in their approach. In cases of disagreement, WL was involved in the decision-making process to resolve discrepancies and ensure the analysis remained consistent.

Through theoretical sampling, data collection, coding, and theory construction were integrated into an ongoing iterative process. A map of expanded theoretical concepts and their connections was developed following the interviews. Theoretical coding played a critical role in clarifying and reinforcing the concepts related to turnover decision-making among nurses with master's degrees.

Since both the interviews and analysis were conducted in Chinese, two bilingual researchers (N.X. and W.W.) independently translated the Chinese analysis results into English. The two translations were cross-checked, and discrepancies were resolved through discussion to reach a consensus version. This consensus version was then verified by a medical English expert with a clinical nursing background (YW, with over 10 years of experience in medical translation and at least five published nursing SCI papers) to ensure accuracy in terminology. This process was intended to guarantee the precision of the results postanalysis [44].

### 2.5. Reflexivity

Data collection was conducted by three researchers—N.X. and W.W. (both holding master's degrees) and WL (with a bachelor's degree), all of whom were experienced in qualitative research and had undergone systematic training in qualitative methodologies. N.X. and W.W., as nurses familiar with the structure, daily operations, and the specific circumstances faced of Chinese hospitals, were considered “insiders.” Their shared professional experiences enabled them to establish a strong rapport with participants, reducing potential emotional disconnect and misconceptions. This insider perspective allowed for a comprehensive exploration and reconstruction of the real-world experiences of the nurses.

However, being insiders also carried the risk of introducing bias. Their personal viewpoints and expectations might have unintentionally influenced the direction of the conversations or shaped the interpretation of certain responses. To minimize this potential bias, N.X. and W.W. made conscious efforts to remain neutral during the interviews, avoiding leading questions and prioritizing participants' perspectives.

Additionally, WL, a nurse without a master's degree and considered a “partial insider,” was included in both the interview process and data analysis to achieve a more objective and well-rounded analysis. Her dual perspective as both a partial insider in the nursing profession and an outsider in terms of educational background facilitated stronger rapport with the participants and offered a counterbalance to the biases of N.X. and W.W.

While WL's involvement contributed valuable objectivity and perspective, her partial insider status also had potential limitations. The shared understanding of nursing practices could have led to subtle assumptions or biases, albeit less pronounced than those typically associated with an entirely insider perspective. To maintain rigor and reflexivity in the study, these potential influences were addressed transparently. Regular team discussions, reflective analysis, and critical engagement with the data were conducted throughout the research process, ensuring that the findings remained as objective and rigorous as possible.

Furthermore, the data regarding turnover rates of nurses with master's degrees in the introduction primarily came from a small-scale survey conducted by the researchers, lacking official statistical support. To address this limitation, this study cross-verified the data with relevant departments and highlighted the source and limitations of the data in the sections where it was referenced. After the data analysis, the results were cross-checked by all co-authors to minimize errors and enhance the credibility and confirmability of the findings [44].

### 2.6. Strategies for Ensuring Research Rigor

To enhance the rigor of this study, several strategies were employed to ensure the credibility, transferability, dependability, and confirmability of the findings [45]. Interviews with nurses across different hospitals and departments were conducted, providing a comprehensive view of the turnover decision-making. Sufficient time was spent engaging with participants to gain a deep understanding of their work environments, a practice that has been shown to improve data credibility. Detailed descriptions of the context, participants, and processes enabled readers to assess the applicability of the findings to other settings, supporting the study's transferability. Additionally, records of all research activities, including data collection, coding, and decision-making processes, were kept to ensure the dependability and replicability of the study. In terms of translation and review, two researchers translated the Chinese version of the analysis and some original data into English, with proofreading by medical English experts to ensure accuracy and dependability.

### 2.7. Ethical Approval and Informed Consent

Ethical approval for this study was granted by the Medical Ethics Committee of Shanxi Bethune Hospital (YXLL-2023-145).

Given the sensitive nature of assessing turnover intention, participants were fully informed about the study's purpose and the voluntary nature of their involvement. Written informed consent was obtained prior to completing the Turnover Intention Scale, with assurances that their responses would remain confidential and be used solely for research purposes. The assessment was conducted with respect and transparency to ensure participants' comfort and understanding of the process.

Participants meeting the inclusion criteria were provided with comprehensive information regarding the study's objectives, procedures, potential risks, and benefits by the interviewer. Informed consent was obtained from each participant before data collection commenced. Participants had the option to provide consent either in writing or verbally, based on their preference. Regardless of the method, the consent process emphasized their rights, the voluntary nature of participation, and the ability to withdraw at any time without consequence. Additionally, measures to protect confidentiality, such as using coded identifiers rather than names, were clearly communicated.

## 3. Results

Eighteen participants were recruited, all nurses with master's degrees from tertiary hospitals across various regions of China, including Shanxi, Shaanxi, Shanghai, Shandong, and Tianjin. Of these, eight nurses had voluntarily submitted their turnover instances within the first two years of employment, while ten were still employed but exhibited high turnover intention (ranging from 14 to 19 points). The group comprised 17 females and 1 male, with ages ranging from 27 to 43 years, and tenure at the hospitals ranging from 0.7 to 13 years. One participant held the position of head nurse, two were officers, and the remaining 15 were or had been frontline clinical nurses. Employment types included permanent (*n* = 3), contract (*n* = 14), and labor dispatch (*n* = 1). Data saturation was reached at the 18th interview. General information about the participants is provided in Table 1.

**Figure 1:**
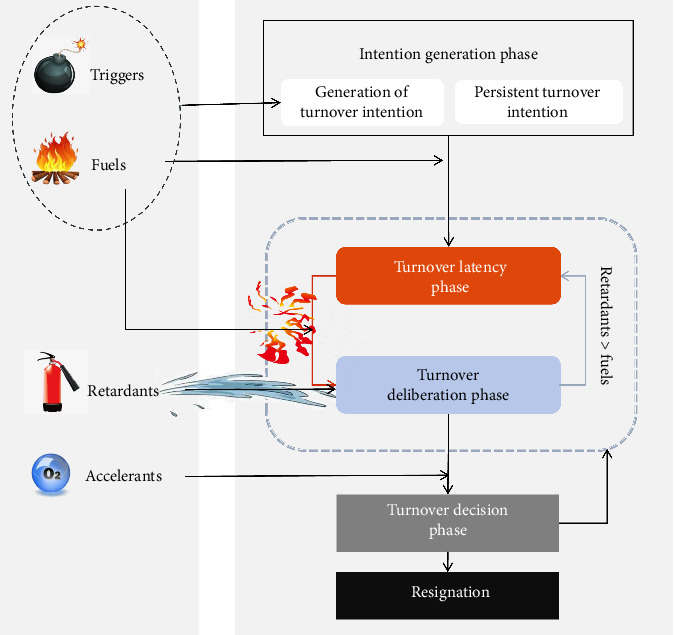
Process from turnover intention to turnover decision for nurses with master's degree.

The study identified four key elements—triggers, fuels, retardants, and accelerants—that influence the process from turnover intention to turnover decision among nurses with master's degrees. This process unfolds in four stages: the intention generation phase, turnover latency phase, turnover deliberation phase, and turnover decision phase, as illustrated in Figure 1.

### 3.1. Elements Influencing the Turnover Decision

#### 3.1.1. Triggers

Triggers are defined as sudden or accidental factors that directly prompt turnover thoughts among nurses with master's degrees. In this study, triggers include personal time exploitation, excessive criticism and education, perceived unfairness, sudden changes in the work environment or relationships, the allure of an ideal job, the influence of colleagues' turnover intentions, unmet promotion expectations, and physical discomfort or illness. These triggers initiate turnover thoughts among nurses.

#### 3.1.2. Fuels

Fuels refer to deep-seated factors that drive nurses with master's degrees to consider turnover. These factors are often difficult to alter in the short term and tend to be insurmountable. Fuels in this study are categorized into traditional and new types. Traditional fuels include persistent inner dissatisfaction, an incompatible department atmosphere, unresolved interpersonal conflicts, overwhelming workloads, disproportionate job compensation, unpredictable professional risks, persistent subhealth conditions, challenges in balancing family obligations, and unmet personal values. These factors commonly influence nurses' turnover decisions.

New types of fuels are more specific to nurses with master's degrees and include insurmountable research demands, diminished personal life, unrecognized psychological pressures, the lingering influence of academic credentials, inexperienced hierarchical management, uncertain career development, and rigid class distinctions between professions.

#### 3.1.3. Retardants

Retardants are factors or events that inhibit the further development of turnover intentions during the decision-making process. These elements serve to neutralize, suppress, or counterbalance turnover inclinations. External factors such as a satisfactory salary, valuable employment opportunities, adjustments to the nature of work, increasing age, and favorable medical conditions act as retardants.

Internal, subjective feelings also play a significant role in discouraging turnover decisions. Retardants, therefore, also include factors such as a strong attachment to original intentions, a resilient personality, a sense of belonging to the department, professional value, institutional pride, fear of change, lack of courage to start anew, shame associated with planning to leave, and uncertainty about life post-turnover.

#### 3.1.4. Accelerants

Accelerants are factors that prompt nurses to make the turnover decision, though they are not the primary causes of turnover. In this study, accelerants include the sudden availability of job opportunities, the unique demands of the profession, role conflicts between professional and personal responsibilities, changes in partner relationships, understanding and support from family, disruptions caused by the COVID-19 pandemic, and emotional support from peers. These factors strongly motivate nurses to decide to turnover.

**Figure 2:**
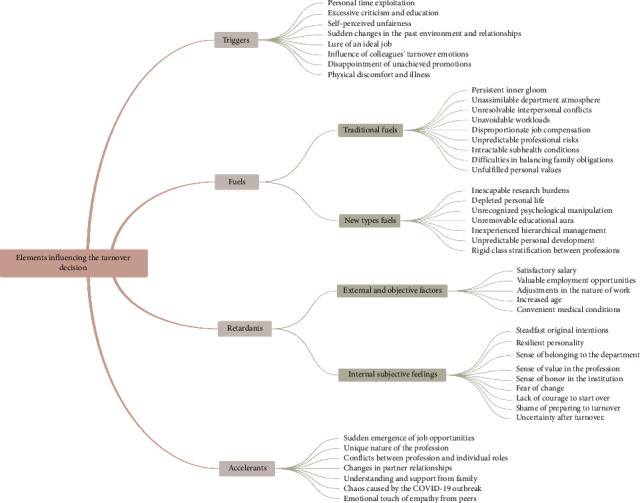
Factors influencing turnover decisions among nurses with master's degree.

The specific factors influencing turnover decisions are detailed in Figure 2.

### 3.2. Process From Turnover Intention to Turnover Decision

This study developed a process model outlining the progression from turnover intention to turnover decision among nurses with master's degrees, consisting of four stages: the intention generation phase, turnover latency phase, turnover deliberation phase, and turnover decision phase. This model is influenced by triggers, fuels, retardants, and accelerants and reflects a complex, narrative-driven process.

#### 3.2.1. Intention Generation Phase

The intention generation phase marks the initial stage of the turnover decision-making process, encompassing both persistent turnover intention and the development of such intentions. Nurses who initially entered the profession due to employer pressure often experience a continual intention to leave the field. Although some may eventually adapt to their workplace, the thought of quitting lingers in varying degrees characterized by a constant state of alertness and readiness to act.

“The hospital was my second choice. After working for a period of time, I found I disliked hospital work. So, I plan to find another job before resigning.” (Participant N2)

“I wanted to be a school teacher at first, but I failed. After entering the hospital, the idea of leaving is still there, but I'm waiting to see if there's anything at the hospital that would entice me to stay.” (Participant N1)

In contrast, other nurses who initially had expectations and confidence in the nursing profession developed turnover tendencies due to the emergence of “triggers” and/or the influence of “fuels.” These factors sparked a desire for a more regular work schedule and a more exciting lifestyle, leading to feelings of exhaustion, doubt, avoidance, or rejection of their current job status.

“I wanted a job that would allow me to take weekends off and be closer to home. That's when I started questioning whether nursing was the right job for me.” (Participant N5)

“I had been steadfast in my decision to work on the clinical front line, but the first time I considered leaving was because of overwhelming work pressure. I'm disappointed by the leadership's inaction. Additionally, several master's colleagues left, and I began to question my choice and my persistence.” (Participant N13)

#### 3.2.2. Turnover Latency Phase

Following the emergence of turnover intention, if the nurse's work situation remains unchanged and the influence of “fuels” continues, the process advances to the second stage: the turnover latency phase. The impact of these fuels is widespread and enduring, causing the thoughts, emotions, and images of wanting to leave the current job not only to persist but also to intensify. Some nurses begin waiting for opportunities or actively seeking out specific job openings. This phase is characterized by an increasingly strong desire to turnover.

“The idea of leaving is always there. Every time I work overtime until 10 p.m., I feel like leaving. My family says that what I say when I get up every morning is that I don't want to go to work.” (Participant N1)

“In the obstetrics department, there's no standardized teaching or training after entering, and the learning resistance is particularly high. My enthusiasm and confidence are draining. Working 12-hour shifts without a break, I don't really want to continue.” (Participant N14)

#### 3.2.3. Turnover Deliberation Phase

For nurses with master's degrees who have entered the turnover latency phase, the influence of fuels gradually intensifies their turnover intention to an unsustainable level, prompting the entry into the third stage: the turnover deliberation phase. During this phase, whether or not new job opportunities have been found, nurses repeatedly weigh the pros and cons of leaving, resulting in internal indecision. This is when retardants emerge, temporarily reducing or weakening their turnover intention, and prompting them to reconsider their decision.

“When I was thinking about leaving, I was hesitant. Because I earn a good income and have made many good friends, I would be reluctant to leave.” (Participant N1)

“The decision to leave was a struggle. At that time, work and life were on the right track, and resignation would require a major change and a fresh start. It was really a rare opportunity to work in a hospital.” (Participant N5)

If retardants can effectively neutralize, cover, or suppress the influence of fuels, some nurses may cycle back from the turnover deliberation phase to the turnover latency phase, delaying the turnover decision. When they encounter the fuels again, their strong turnover intention is reactivated, propelling them back into the turnover deliberation phase. If the retardants fail to counteract the fuels, the turnover decision advances to the next stage.

“At that time, I really wanted to leave. After weighing the pros and cons, I felt that I couldn't give up the status of a regular employee and the high salary.” (Participant N17)

“I feel sick after working the night shift. The idea of resignation was particularly strong. But when the intensity of the work eased a bit and the physical pressure lessened, especially with the good pay, I felt I could hang on for a while.” (Participant N12)

“Although the salary and platform at the hospital are very high, I really can't bear being oppressed in the hospital for a lifetime, so I resigned after weighing it all up.” (Participant N2)

#### 3.2.4. Turnover Decision Phase

When nurses are in the process of weighing whether to turnover, the emergence of unforeseen opportunities or sudden events (the accelerants) can propel them to make a final decision, advancing the process to the fourth stage—the turnover decision phase. This phase is characterized by a shift from complex internal struggles to a firm conviction, with the turnover decision typically becoming resolute. In the absence of accelerants, some resilient nurses may delay or suppress their turnover intentions, or these intentions may diminish over time, resulting in a temporary suspension of the decision.

“There were some job postings that I was interested in. My family and I made long-term plans, and with their support, I resigned without hesitation and started preparing for the exam.” (Participant N1)

“At that time, I applied for a position in another unit, planning to resign once I found a new job. However, I didn't get the position, and no other suitable opportunities arose, so the resignation was temporarily put on hold.” (Participant N9)

### 3.3. Substantive Theory Development

The developed substantive theory in this study is titled “The Process of Turnover Decision-Making Among Nurses with Master's Degrees.”

#### 3.3.1. Components of the Substantive Theory

##### 3.3.1.1. Phases of Turnover Decision-Making

Intention Generation Phase: The emergence and persistence of turnover thoughts are initially triggered and sustained by external and internal factors.

Turnover Latency Phase: Strengthening of turnover intentions is influenced by the continued effects of these factors on perceptions and emotions.

Turnover Deliberation Phase: A careful evaluation of the advantages and disadvantages is shaped by deterrents that temporarily suppress or neutralize turnover intentions.

Turnover Decision Phase: The final decision is often solidified by accelerants that reinforce the intent to leave.

##### 3.3.1.2. Elements Influencing Turnover

Triggers: Abrupt factors that spark the consideration of turnover.

Fuels: Underlying issues that sustain and intensify the desire to leave.

Retardants: Elements that momentarily diminish turnover intentions.

Accelerants: Events or opportunities that prompt the final turnover decision.

##### 3.3.1.3. Contextual Factors

Personal and Professional Contexts: These shape the way nurses perceive and respond to triggers, fuels, retardants, and accelerants.

Organizational Dynamics: Workplace policies, culture, and interpersonal relationships influence turnover intentions.

#### 3.3.2. Contribution to Grounded Theory

This theory integrates qualitative data into a cohesive framework that captures the dynamic process of turnover decision-making among nurses with master's degrees. It provides valuable insights into the interactions and progression of various factors, ultimately explaining how turnover intentions may either persist or be resolved over time.

## 4. Discussion

### 4.1. Elements Influencing Turnover Decisions Among Nurses With Master's Degrees

The turnover decision is a dynamic, multistage process shaped by factors across multiple levels [23, 46]. Employing grounded theory, four critical elements—triggers, fuels, retardants, and accelerants—were identified as central to the decision-making framework.

We found that triggers and accelerants, such as the ripple effects of colleague turnover, unmet promotion expectations, or changes in personal relationships, primarily operate through specific scenarios or abrupt events that initiate and propel the turnover process. This aligns with the findings of Nei et al. [47] and Chang et al. [48]. However, whether these elements provoke turnover decisions largely depends on an individual nurse's experiences and interpretation of external events, which shape their self-perception and attitude. Each unique or sudden event carries its own context, and different nurses interpret these events through the lens of their personal cognition and social background [30, 49]. Thus, while predicting individual reactions to such events is challenging, the occurrence of these events should alert nursing managers to take proactive steps to mitigate turnover thoughts among nurses.

In contrast to triggers and accelerants, fuels and retardants encompass a broader range of factors and are the primary determinants of turnover decisions among nurses with master's degrees. The fuel factors identified in this study include workplace atmosphere, interpersonal relationships, workload, compensation, health status, family responsibilities, and self-worth. These factors closely align with findings from previous studies on turnover among nurses without master's degrees [25, 50]. The International Council of Nurses has highlighted common turnover drivers across countries, such as poor working conditions and the failure to implement safe staffing levels [16]. These pervasive challenges in the nursing environment, termed “traditional fuels,” are critical to nurse retention worldwide [16]. Consequently, the findings of this study may be applicable to other healthcare systems, although specific challenges may vary based on local cultural and organizational contexts.

However, this study also found that nurses with master's degrees are influenced by additional factors beyond these traditional challenges, which further drive their turnover intentions.

### 4.2. Emerging Fuels in Turnover Decision of Nurses With Master's Degrees

This study identified new types of fuels, including inescapable research burdens, a depleted personal life, the persistent influence of academic qualifications, and inexperienced grassroots management, as unique factors in the turnover decision process of nurses with master's degrees. The authors believe that the underlying causes of these negative emotional experiences can be attributed to three primary factors: self-positioning, educational background, and hospital management.

#### 4.2.1. Mismatched Career Expectations and Clinical Realities for Nurses With Master's Degrees

Long-term career perspectives are shaped by factors such as age, years of experience, personal traits, upbringing, and economic status [26, 51]. However, our study found that many nurses pursuing master's degrees in nursing in China often have a limited understanding of the professional positioning and employment prospects for master's degree holders. They tend to assume that, due to their advanced education and its relative rarity, master's degree nurses will easily secure comfortable, prestigious, and well-compensated positions. This expectation leads to high career aspirations and a sense of prestige tied to their qualifications. However, the university training model, which emphasizes theory over clinical practice and research over practical application, does not align with the healthcare sector's demand for highly skilled clinical nurses [27]. This misalignment causes many nurses to feel undervalued, unable to fully utilize their skills, or even diminished in their clinical roles, ultimately resulting in confusion or disillusionment regarding their careers [52].

To address this gap, it is recommended that universities strengthen their social services to provide nursing students with a clearer understanding of the current social and industry landscape. Such initiatives will help students better adapt to the workforce, foster a stronger sense of professional mission, and bridge the divide between academic education and the practical demands of the healthcare sector [53].

#### 4.2.2. Challenges in Integrating Academic Knowledge With Clinical Practice

Nurses with master's degrees often possess advanced skills in information gathering, research, computer literacy, and critical thinking. However, they face significant challenges in integrating academic knowledge with clinical practice, leading to frustration [8, 9]. The disconnect between theoretical expertise and practical application undermines their effectiveness in clinical settings. Moreover, in the context of human resource shortages and heavy workloads, these nurses are burdened with additional research and departmental responsibilities. The resulting overload, coupled with the prolonged development process required for advanced nursing talent, limits personal growth and immediate rewards [28].

To address these challenges and achieve the goals of retaining highly skilled nursing talent, stabilizing the nursing workforce, and enhancing the scientific rigor of nursing work, this study recommends the development of a collaborative training model that involves universities, society, and hospitals. This model should focus on clinical demands and define the orientation of advanced, applied, and specialized training for professional nursing master's programs. Furthermore, it should rigorously regulate the clinical practice components and refine the training process for master's degree nurses [54].

#### 4.2.3. Impact of Leadership Styles on Nurses With Master's Degrees

As the foundational leadership tier in medical organizations and the direct managers of nurses, the leadership style of head nurses profoundly affects the work environment, departmental culture, and organizational cohesion [55, 56]. This study identifies factors such as discordant departmental atmosphere, excessive criticism, and psychological manipulation as key elements that influence turnover intentions among nurses with master's degrees. These factors are directly linked to the leadership style of grassroots managers, aligning with the findings of Türkmen et al. [57]. Additionally, the relatively low proportion of nurses with master's degrees in China (ranging from 0.1% to 0.3%) [14] suggests that grassroots managers often lack experience in managing these highly educated nurses. This gap is evident in areas such as task allocation, performance evaluation, salary distribution, professional development, organizational structure, and career expectations [8, 58]. The misalignment between initial employment expectations and current professional realities creates a significant disconnect for nurses with master's degrees, making it difficult for them to reconcile their situation and leading to internal conflict [8, 9, 59].

To address this, nursing administrators should refine their management strategies for nurses with master's degrees by considering both their personal circumstances and professional positioning [60]. Implementing magnet nursing management principles is crucial to fostering a positive, harmonious, and supportive work environment for these highly educated nurses.

### 4.3. Retardants in Turnover Decision of Nurses With Master's Degrees

Furthermore, factors that prevent the escalation of turnover intentions are categorized as retardants, including a steadfast original intent, a resilient personality, a sense of institutional loyalty, fear of change, and a lack of courage to start anew. These internal factors inhibit turnover decisions, which aligns with the findings of Zhu et al. [52]. Nursing administrators should focus on clarifying professional roles, instilling professional values, and addressing negative experiences to mitigate turnover intentions.

Our study also indicates that while increasing age may superficially hinder turnover decisions, the underlying factor is generational differences. As noted by Fang et al. [61], different generations of nurses possess distinct work values, communication styles, and expectations regarding leadership and work environments. Xiong et al. [62] further emphasized that the newer generation of knowledge workers prioritizes organizational atmosphere, advocates for a more flexible lifestyle, and values work–life balance. Therefore, it is recommended that nursing administrators understand the generational characteristics of nurses with master's degrees and develop strategies to attract, motivate, and retain this new generation of professionals.

### 4.4. Professional Hierarchies and Turnover Decisions Among Nurses With Master's Degrees

The solidification of class hierarchies between professions emerged as a key factor in our study, revealing a notable yet surprising trend. This concept refers to a hierarchical culture between doctors and nurses, where nurses with master's degrees perceive themselves as subordinate to doctors. Furthermore, the treatment of nurses with master's degrees by medical units significantly differs from that afforded to doctors with similar qualifications [63]. This professional stratification not only influences nurse–doctor interactions but also plays a pivotal role in the turnover decisions of nurses with advanced degrees [64]. Despite ongoing challenges, barriers to full partnership between nurses and physicians persist.

The sense of subordination to doctors has primarily been reported in studies conducted in Iran, Finland, and China [65–67]. This suggests that the entrenchment of professional hierarchies is deeply influenced by cultural and socioeconomic contexts. In China, the hierarchy may stem primarily from nurses' limited decision-making autonomy. The rapid rise in patient numbers, coupled with a severe nursing shortage [68], has led nurses to operate under chronically overburdened conditions, fostering dependency on doctors' diagnoses and decisions. As a result, nurses' abilities in assessment, diagnosis, intervention, and critical thinking have diminished [69, 70], even among those with master's degrees. Thus, while advocating for the dissolution of the medical-nursing hierarchy, it is essential that nurses with advanced degrees also focus on enhancing their professional competence and independent decision-making skills, thereby assuming a leadership role in nursing assessment, diagnosis, planning, and policy development.

### 4.5. Understanding the Turnover Decision Process Among Nurses With Master's Degrees

This study constructed a decision-making process for turnover among nurses with master's degrees. A subset of these nurses exhibits a persistent propensity for turnover from the outset of their employment, maintaining a state of heightened vigilance and readiness for new opportunities. This suggests that premature or hasty career choices may pose a significant risk for turnover among nurses with advanced degrees [71]. Hospitals are encouraged to gain a deeper understanding of these nurses' career goals and aspirations during recruitment, utilizing tools such as the Occupational Identity Scale and Turnover Intention Scale to identify candidates with strong professional commitment [9]. Another group of nurses with master's degrees develops turnover intentions due to the influence of specific triggers (accidental or sudden events) and/or underlying fuels (fundamental, hard-to-alter factors). These elements generate negative feelings toward their current roles, leading to a shift in mindset and the emergence of turnover intentions.

Under the influence of these fuel factors, all nurses with master's degrees, regardless of their specific circumstances, become alert and begin to consider turnover, transitioning from the initial phase of intention formation to turnover latency. As the influence of fuel elements persists, turnover intentions continue to intensify. However, “retardants” emerge during the turnover deliberation phase, prompting nurses to carefully assess the pros and cons of leaving their positions. It is during this phase that managers must identify nurses considering turnover, understand the primary factors driving their decision-making, and strategically apply or enhance the influence of retardant factors to slow down or counteract the turnover process, thereby reducing attrition. When the impact of retardants outweighs that of fuels, the turnover decision may be temporarily postponed. However, should the nurses encounter new fuel elements, their turnover intentions may be reignited, bringing the decision-making process back to the forefront.

Consequently, healthcare institutions must prioritize addressing the core issues driving turnover among nurses with master's degrees, aiming to extinguish the underlying causes and prevent the escalation of turnover [29]. Ultimately, the emergence of “accelerants” leads these nurses to make a final decision, entering the turnover decision phase and resigning. This framework encapsulates the comprehensive process from turnover intention to decision-making among nurses with master's degrees.

### 4.6. Applicability of Findings in Global Healthcare Systems

Although this study focuses on China, turnover intention among nurses with a master's degree is also a growing concern in other countries. Globally, research indicates that APNs experience turnover intention influenced by both intrinsic and extrinsic factors [19]. The study from the United States links job dissatisfaction among APNs to inadequate administrative support, salary concerns, and lack of professional recognition [72]. This mirrors findings in China, where master's degree nurses also identify similar issues as key drivers of turnover intention. However, cultural and organizational differences can affect the weight of these factors [73, 74]. In hierarchical healthcare systems like the United States and United Kingdom, managerial support and workplace communication play a significant role in job satisfaction [75]. In contrast, in more egalitarian systems, such as those in Scandinavian countries, where teamwork and autonomy are emphasized, hierarchical leadership's influence on turnover may be less pronounced [73].

Similarly, our study found that limited opportunities for career advancement, inadequate compensation, and excessive workload are major barriers to job satisfaction and retention among master's prepared nurses. A systematic review found that the intention to leave is often driven by unmet professional needs, both intrinsic and extrinsic, highlighting the critical factors influencing retention among APNs [19]. These parallels underscore the broader relevance of our findings and highlight the universal importance of supportive work environments in retaining highly educated nursing professionals. Furthermore, understanding the transition from turnover intention to turnover decision is crucial for informing international policies aimed at retaining advanced practice nurses.

### 4.7. Strengths and Limitations

Participants of this study are from tertiary hospitals across various regions of China, contributing to geographical diversity. However, the exclusive focus on tertiary hospitals may limit the generalizability of the findings to other healthcare settings, such as secondary hospitals, community health centers, or nursing homes. Therefore, the results of this study are specific to the context of provincial tertiary hospitals in China and should not be easily generalized to other regions or healthcare systems.

The research team includes nursing managers and nurses with master's degrees, all of whom have extensive clinical and managerial experience. This background enables a deeper understanding of, and empathy with, participants' working environments. However, such professional credentials may also cause interviewees to feel cautious or reluctant to discuss sensitive issues. These strengths and limitations are comprehensively addressed in the Reflexivity section of this study. Therefore, the potential influence of the researchers' background should be interpreted within this broader analytical framework.

Additionally, the inclusion of only one male nurse reflects the challenges in recruiting male nurses, particularly given their low representation among nurses with master's degrees. This underrepresentation may affect the generalizability of the findings, particularly with regard to gender-specific challenges or attitudes. Future research should examine factors deterring male participation and explore strategies such as targeted recruitment and the creation of a more inclusive workplace culture to boost male nurse representation, thereby offering a more comprehensive understanding of gender dynamics in nursing.

## 5. Conclusion

This study employed grounded theory to investigate the turnover decision-making process among Chinese nurses with master's degrees, developing a framework consisting of triggers, fuels, retardants, and accelerants to map the stages from turnover intention to decision.

The findings highlight the internal conflicts and contextual factors affecting nurses with master's degrees, revealing how academic qualifications, management styles, and professional hierarchies significantly influence turnover decisions. These insights can help nursing managers craft targeted strategies for each stage, thereby mitigating turnover risk and improving retention.

Future research could explore the impact of external factors, such as healthcare policies, economic conditions, and global trends, on turnover dynamics among highly educated nurses. Longitudinal studies tracking the career paths of nurses with master's degrees would provide valuable insights into the long-term effects of turnover decisions. Additionally, comparative studies across different healthcare environments and cultural contexts could clarify the universal and context-specific factors influencing turnover.

## 6. Implications for Nursing Management

This study provides stakeholders and policymakers with insights to proactively address and mitigate turnover among nurses with master's degrees. Nursing managers can identify and track the progression of turnover intention through the four stages from intention to decision. Tailored management strategies should focus on key influencing factors, strengthening protective factors that impact turnover decisions, rebuilding nurses' confidence in continued employment, and reducing both turnover intention and overall turnover rates. These insights lay a theoretical foundation for refining talent development programs and implementing effective management strategies.

## Figures and Tables

**Table 1:** Participants' general information and turnover status.

N	A	G	T	L	D	PT	P	ME	MS	C	LHS (year)	LT (month)	TIS	IF
N1	28	Female	Academic master	Shaanxi	Operating theatre	Nurse practitioner	Nurse	Contract worker	Married	0	2	2	NA	Online
N2	28	Female	Professional master	Shanxi	Department of Gastrointestinal	Nurse practitioner	Nurse	Contract worker	Unmarried	0	1	13	NA	Online
N3	27	Female	Academic master	Shanxi	Department of Orthopedics	Nurse practitioner	Nurse	Contract worker	Unmarried	0	1.3	11	NA	Online
N4	32	Female	Professional master	Shanxi	Department of Pneumology	Nurse-in charge	Nurse	Contract worker	Unmarried	0	1.6	5	NA	Online
N5	28	Female	Academic master	Shanxi	Department of Geriatrics	Nurse practitioner	Nurse	Contract worker	Unmarried	0	1.08	12	NA	Online
N6	27	Female	Academic master	Shaanxi	Department of Geriatrics	Nurse practitioner	Nurse	Contract worker	Unmarried	0	0.7	5	NA	Online
N7	27	Female	Professional master	Shanghai	Department of Nursing	Nurse practitioner	Research officer	Labor dispatching	Unmarried	0	1.5	8	NA	Online
N8	30	Female	Academic master	Tianjin	Department of Neurology	Nurse practitioner	Nurse	Contract worker	Married	0	4	6	NA	Online
N9	27	Female	Professional master	Shanxi	Operating theatre	Nurse practitioner	Nurse	Contract worker	Unmarried	0	2	NA	15	Face to face
N10	30	Female	Academic master	Shanxi	Department of Oncology	Nurse practitioner	Nurse	Contract worker	Unmarried	0	2	NA	17	Online
N11	43	Male	Professional master	Shanghai	Operating theatre	Co-chief superintendent nurse	Head nurse	Permanent employee	Married	1	13	NA	17	Face to face
N12	28	Female	Academic master	Shandong	Department of Neurology	Nurse practitioner	Nurse	Permanent employee	Married	0	2	NA	14	Online
N13	28	Female	Academic master	Shanxi	Department of Gastrointestinal	Nurse practitioner	Nurse	Contract worker	Unmarried	0	2	NA	15	Face to face
N14	28	Female	Academic master	Shanxi	Department of Delivery Room	Nurse practitioner	Nurse	Contract worker	Unmarried	0	2	NA	14	Face to face
N15	28	Female	Academic master	Shanxi	Department of Cardiac Surgery	Nurse practitioner	Nurse	Contract worker	Unmarried	0	2	NA	19	Face to face
N16	29	Female	Professional master	Shanxi	Department of Urinary Surgery	Nurse practitioner	Nurse	Contract worker	Married	0	3	NA	18	Online
N17	38	Female	Academic master	Shaanxi	Department of Nursing	Co-chief superintendent nurse	Nursing officer	Permanent employee	Married	1	12	NA	14	Face to face
N18	30	Female	Academic master	Shanxi	Department of Thoracic Surgery	Nurse-in charge	Nurse	Contract worker	Married	1	4	NA	18	Face to face

*Note:* A, age; C, number of children; D, department; G, gender; L, location of the working hospital; LHS, length of hospital service (onset resignation); N, number; P, posts; T, types of master's degree.

Abbreviations: IF, interview form; LT, length of turnover; ME, method of employment; MS, marital status; NA, not applicable; PT, professional title; TIS, Turnover Intention Score.

## Data Availability

Data used to support the findings of this study are available from the corresponding author upon reasonable request.
